# Retrospective evaluation of referral by community health workers on the uptake of intermittent preventive treatment of Malaria in pregnancy in Ohaukwu, Ebonyi State, Nigeria

**DOI:** 10.1186/s12884-022-04921-7

**Published:** 2022-07-27

**Authors:** Bright Chukwudi Orji, Emily Bryce, Bartholomew Odio, Herbert Enyeribe Onuoha, Elizabeth Njoku, Charity Anoke, Emmanuel Ugwa, Joseph Enne, Adetiloye Oniyire, Emmanuel Otolorin, Kayode Afolabi, Nnenna C. Ogbulafor, Elizabeth Oliveras

**Affiliations:** 1 Jhpiego - an Affiliate of Johns Hopkins University, Abuja, Nigeria; 2 Jhpiego - an Affiliate of Johns Hopkins University, Baltimore, MD USA; 3 Federal Medical Center, Brinin Kudu, Nigeria; 4 Reproductive Health Division, Federal Ministry of Health, Abuja, Nigeria; 5 National Malaria Elimination Program, Federal Ministry of Health, Abuja, Nigeria

**Keywords:** Intermittent preventive treatment, Malaria, Pregnancy, Community distribution, Antenatal care

## Abstract

**Background:**

The World Health Organization recommends a minimum of three doses of quality-assured sulfadoxine-pyrimethamine for intermittent preventive treatment of malaria during pregnancy (IPTp), in moderate to high malaria transmission areas in sub-Saharan Africa. Currently, coverage of IPTp lags behind coverage of antenatal care (ANC) visits; in Nigeria, 57% of women attended four or more ANC visits, whereas only 17% received the recommended three or more doses of IPTp. The innovative program aimed to close this gap by providing counseling on the benefits of comprehensive ANC, referral to ANC and community distribution of IPTp (C-IPTp), complementing IPTp at ANC. The study aimed to examine whether CHW referral to ANC improved the likelihood of receiving three or more doses of IPTp.

**Methods:**

The data for this study were extracted from the maternity record books of 1437 pregnant women seen at 25 public health facilities in Ebonyi State, Nigeria. The outcome of interest was defined as the receipt of three or more doses of IPTp (IPTp3) and the independent variable was referral to ANC by a community health worker for any visit. Descriptive statistics were reported and the results from the multi-level regressions are reported as adjusted odds and prevalence ratios with corresponding 95% confidence intervals.

**Results:**

Of the 936 women included in the analysis, 24.47% received the recommended three or more IPTp doses and 61.32% were referred by a community health worker (CHW) for at least one ANC visit. There was no difference in the mean number of ANC visits between women who received C-IPTp and those who received IPTp exclusively at a facility (2.40 vs 2.52; *p* = 0.374). There were no maternal characteristics associated with CHW referral. Women who were referred by a CHW had 60% greater odds (95% CI, 1.08–2.38) of receiving IPTp3 than those who were never referred.

**Conclusion:**

The results indicate that CHW referrals conducted within a C-IPTp program are associated with higher IPTp uptake but not fewer ANC visits and that CHWs applied the referral process equally. This strengthens the evidence base for C-IPTp scale-up, which could have a large impact in sub-Saharan Africa in mitigating existing health systems issues.

**Supplementary Information:**

The online version contains supplementary material available at 10.1186/s12884-022-04921-7.

## Background

The World Health Organization (WHO) recommends a minimum of three doses of quality-assured sulfadoxine-pyrimethamine (QA SP) for intermittent preventive treatment of malaria during pregnancy (IPTp) beginning as early as possible in the second trimester. Reducing malaria in pregnancy contributes to decreased incidence of low-birth-weight babies by 21%, severe maternal anemia by 40% and neonatal mortality by 18% [1, 2]. IPTp is an intervention specifically for moderate to high transmission areas in sub-Saharan Africa (SSA), in addition to standard malaria prevention interventions including use of insecticide-treated bed nets (ITNs) and effective case management. According to the 2021 World Malaria Report, in 2020 in SSA it is estimated that only 32% of pregnant women received the WHO-recommended three or more doses [3] of IPTp, despite almost half of pregnant women attending antenatal care (ANC) four times. These data suggest delivering IPTp via ANC alone is insufficient to achieve the WHO recommendation. This trend holds true in Nigeria as well, where 57% of women attended four ANC visits, but just 17% received three or more doses of IPTp [4].

Building on prior success engaging community health workers (CHWs) to deliver health interventions, including distribution of IPTp [5–9], the Transforming Intermittent Preventive Treatment for Optimal Pregnancy (TIPTOP) project was launched by the Ohaukwu local government area (LGA) of Ebonyi state in September 2018. TIPTOP provided eligible pregnant women more opportunities to receive IPTp, both close to their homes through a network of purposely trained and supervised, easily accessible CHWs, and at ANC. In this approach, called community-IPTp (C-IPTp), CHWs promoted ANC attendance by counseling pregnant women on the benefits of comprehensive ANC and referring them to ANC, while also providing IPTp. TIPTOP demonstrated C-IPTp can increase coverage of IPTp3 (three or more doses of IPTp) without negatively affecting ANC attendance.

This study examined whether CHW referral to ANC improved the likelihood of receiving IPTp3 and if any additional maternal characteristics were associated with IPTp3 uptake. Ultimately, the aim was to better understand the role of ANC referrals as part of the innovative C-IPTp strategy and its association with IPTp3 to guide future program implementation and provide recommendations on the use of CHWs to increase uptake of IPTp.

## Methods

### Study area

The study was conducted in Ohaukwu LGA, one of the thirteen LGAs of the southeast state Ebonyi. The LGA has one secondary hospital and 51 primary health care centers (39 public and 12 private) that provide ANC services. This rural LGA has an estimated area of about 517 km^2^, with a population of 294,179 (2020) comprised primarily of subsistence farmers. Ohaukwu has a tropical climate divided into two seasons – wet and dry – with an average annual rainfall of 1300 mm.

### Study population

The study population included pregnant women who enrolled in ANC services at 25 health facilities in Ohaukwu LGA between April and September of 2019*.* Private facilities were excluded because there were too few to analyze the data separately. Additionally, several sites from Effium zone were unable to be included due to communal clashes during the time of data collection. Stratified random sampling was used to select the 25 health facilities from a sampling frame of the 42 TIPTOP supported, CHW-linked public health facilities stratified by antenatal client volume to reflect the large variability in the number of women registered for ANC at each site. Data were captured for all women seen during the study period and who had a Maternity Record Booklet (MRB) on file. Women were excluded from the analysis if they registered for their first ANC visit less than 8 weeks before their delivery (either because they registered late or because they had a preterm birth) or if they were taking medications that contraindicated IPTp.

### Data source

The data for this cross-sectional study were extracted from the MRB which is a client-level medical record for pregnancy, labor and delivery, and the postpartum period. The MRB collects individual-level data that is not available in other routine data sources which are reported in aggregate. ANC visits and referrals, IPTp doses and location of administration (community versus ANC), and various maternal characteristics are among the data captured in the MRB. It is completed by the frontline service provider who provides ANC, delivery or postpartum services. Referrals are entered if the woman arrives with a referral slip from a CHW; only TIPTOP CHWs refer women to ANC in the LGA.

A data extraction tool was adapted from a standardized data extraction tool used by the U.S. Zika Pregnancy Registry and Zika Birth Defects Surveillance [10]. It was pretested for clarity, content validity and reliability. The tool was accompanied by an extraction manual, including written extraction instructions, operational definitions, and a description of both where the data elements are located in the MRB and where they were to be entered. The data were entered into a browser-based application, REDCap (Research Electronic Data Capture) hosted securely on a Jhpiego server. The tools were pretested in Abakaliki, an LGA which is outside, but comparable to, the study site. The pretest was conducted immediately after the training of data collectors in July 2021.

### Sample size

We estimated that 79% of women who enrolled in ANC will be eligible to complete IPTp3, given 21% of women attend their first ANC visit in the 6th month of pregnancy or later and that women who presented for care less than 8 weeks before delivery were excluded from the analysis [4]. A feasibility assessment was conducted for this study, which revealed significant variability in the number of women registered for ANC at each facility. Therefore, a simulation study was run which varied the number of facilities (between 20 and 32) to assess the empirical statistical power. The goal was to determine the number of facilities that allowed for 80% power to reject the null hypothesis of no difference in IPTp3 coverage by ANC referral by CHW. For this, 1000 replicates of the study design were generated, each with a specific number of facilities and assuming 0.05 intra-facility correlation of IPTp3 coverage. We assumed a 35% probability of ANC referral and the probabilities of receiving 3 IPTp doses - 50 and 60% for non-referred and referred women, respectively. The number of ANC records per facility was drawn from a uniform distribution with a range between 1 and 75 (based on the feasibility assessment, which showed a range of 1 to 75 women enrolled per month at sampled facilities) to account for large variability and making every number in this range equally possible for each facility. A sample of 25 facilities was associated with 79% empirical statistical power in the simulations. Of the 1473 Maternity Record Booklets from which data were abstracted, a total of 936 were included in the analysis. Records were dropped from the analysis if they did not meet the exclusion criteria (*N =* 182) or were missing data (*N =* 355).

### Variables

The primary outcome of this study was the receipt of three or more IPTp doses during pregnancy (IPTp3). This included both doses received during ANC and in the community. The primary covariate of interest was referral of the pregnant woman to ANC by a CHW; this included referral to *any* visit, not just to the first visit. Maternal age was categorized after examination of the LOWESS (locally weighted scatterplot smoothing) graph into the following age groups: 14–19 years, 20–24 years, 25–29 years, 30–34 years and 35 years old and older. Parity, or the number of times a woman has given birth to a fetus with a gestational age of 24 weeks or greater, was categorized into nulliparous (no previous births), primiparous (one previous birth) and multiparous (two or more previous births). A binary “yes or no” variable for receipt of malaria-related education during ANC was included. Gestational age (in months) at first ANC visit was calculated using this date and the reported date of last menstrual period when available. If not available, the gestational age reported by the pregnant woman at the first ANC visit was used. Data were collected on both marital status and ethnic group, but not included in the models due to a lack of heterogeneity. Educational status and occupation could not be included in the models due to high missingness (> 15% women had missing data).

### Statistical analysis

The analysis was conducted in Stata version 14. Chi-squared tests were used to compare pregnant women 1) who did and did not receive IPTp3 as well as those 2) who attended ANC but never received a dose of IPTp and those that did. Bivariate logistic regressions were used to examine the unadjusted associations between the outcome, IPTp3, and the covariate of interest, CHW referral to ANC, and other possible confounders including maternal characteristics (i.e., maternal age, parity, malaria-related education during ANC and gestational age at first ANC visit).

Generalized linear mixed models were used to evaluate if women who were referred into ANC services by a CHW were more likely to complete the third dose of IPTp (IPTp3) than women who do not receive any referrals. Two multilevel logistic regression models with a random intercept for facility were run; a null model to estimate the proportion of the variation in the outcome attributable to unobserved facility-level characteristics and a model to examine the association’s individual-level characteristics and the outcome, IPTp3 receipt. The random intercept accounts for the clustering at the facility level, whereby women who attend the same facility may be more similar than those who attend another facility and to account for similarities in care provided at a given facility. The variance inflation factor (VIF) to check for multicollinearity, of which there was no evidence (VIF = 1.15).

To examine whether certain characteristics were associated with CHW referral to ANC, two multilevel Poisson models with a random intercept for facility were run (results can be found in full in the supplementary materials). Due to the high prevalence of the binary outcome, ever referred for an ANC visit by a CHW, a multilevel Poisson regression with robust variance estimation and a random intercept for facility was used to calculate adjusted prevalence ratios (aPR). No evidence of multicollinearity was found here either (VIF = 1.22).

### Ethical approval

The study was approved by the Institutional Review Board of the Johns Hopkins Bloomberg School of Public Health, the State Ministry of Health Ebonyi State Research Ethics Committee of Nigeria and World Health Organization Research Ethics Review Committee. All methods in this study were carried out in accordance with the relevant guidelines and regulations of Helsinki Declaration. The study protocol was approved with statement on informed consent as submitted. Informed consent was obtained from all the facility managers that participated in the study to abstract data from their antenatal care registers and maternity record booklets; while informed consent from research subjects was waived by the three approval organizations given that data abstracted and analyzed were collected as part of routine antenatal care, and usual health care services. In addition, no identifying information or sensitive topic were collected.

## Results

**Fig. 1:**
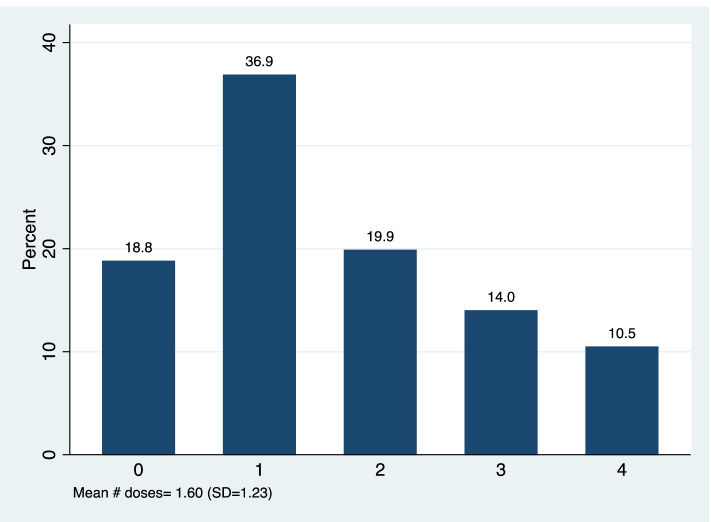
Distribution of the # of IPTp doses

**Fig. 2:**
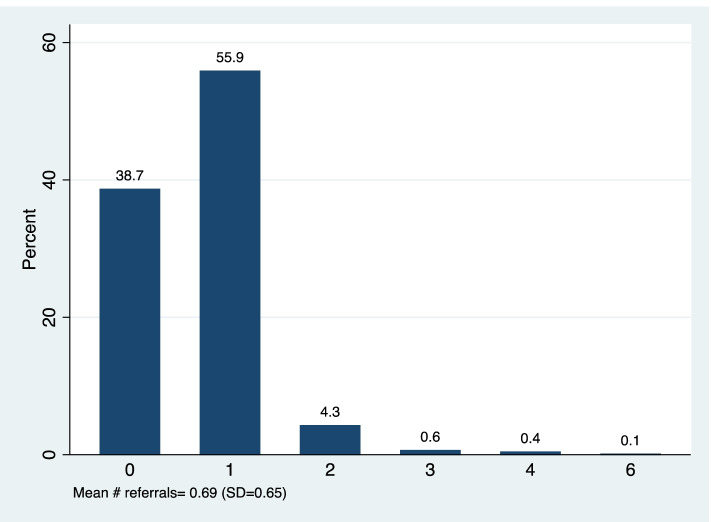
Distribution of the # of referrals for ANC visits

Of the 936 women included in the analysis, the average age was 26.79 years (SD = 5.49) with the youngest woman aged 14 years and the oldest aged 46 years (Supplementary Fig. 1). Nearly 15% of women had never given birth before (Table 1). The majority (64.96%) of women had given birth two or more times. More than half of women presented for their first antenatal care (ANC) visit during their second trimester (58.33%). Only 18.4% of women completed four or more ANC visits, with an average of 2.28 visits (Supplementary Fig. 2). Nearly 90% of women were recorded as receiving malaria-related education during ANC. Finally, nearly one quarter of women were recorded as receiving three or more doses of IPTp and the largest proportion of women received only one dose (36.9%) (Fig. 1). 61.3% of women were referred by CHWs at least once and 5.4% of women received two or more referrals (Fig. 2). There were no characteristics that were significantly associated with CHW referral (Supplementary Table 1).

**Table 1:** Participant characteristics (*N =* 936)

Variable	N	%
**Woman’s age (years)**		
14 to 19 years	64	6.84%
20 to 24 years	252	26.69%
25 to 29 years	327	34.94%
30 to 34 years	189	20.19%
35 years or more	104	11.11%
**Parity**		
Nulliparous	139	14.85%
Primiparous	189	20.19%
Multiparous	608	64.96%
**Malaria education in ANC**		
No	94	10.04%
Yes	842	89.96%
**Trimester at first ANC visit**		
First trimester	260	27.78%
Second trimester	546	58.33%
Third trimester (7th month)	130	13.89%
**Women attended four or more ANC visits**		
No	764	81.62%
Yes	172	18.38%
**Woman was referred by CHW for at least one ANC visit**		
No	362	38.68%
Yes	574	61.32%
**Woman received 3 or more IPTp doses**		
No	707	75.53%
Yes	229	24.47%

Fifty-six percent of women in the study population received at least one IPTp dose from a CHW (note, that for 22% of women in the study population, the location of dose administration was missing in the MRB). We compared the mean number of visits among women who received at least one IPTp dose from a CHW (μ = 2.40, SD = 1.56) versus those that only received IPTp in a facility (μ = 2.52, SD = 1.69). There was no significant difference between the means (*p* = 0.374). Additionally, the proportion of women who attended at least four ANC visits was not different between women who received IPTp at the community from a CHW when compared to those that only received IPTp at ANC (p1 = 0.198 p2 = 0.238, *p*-value = 0.240).

Table 2 presents the number and proportion of women who did and did not receive IPTp3 along with the unadjusted Odds Ratios (OR) and corresponding 95% confidence intervals (95% CI). The unadjusted OR estimates do not account for the clustering of women within facilities. A woman that was referred to at least one ANC visit by a CHW had 1.52 times greater odds (95% CI: 1.10–2.08) of IPTp3 receipt than those who were never referred for ANC by a CHW. The odds of receiving IPTp3 increased with the age of the pregnant women but this was not statistically significant. The odds of receiving IPTp3 were nearly two times greater among women who were provided malaria-related education during antenatal care compared to those that did not (OR = 1.79, 95% CI: 1.01–3.18)). No other covariate had a significant relationship with the outcome.

**Table 2:** Characteristics associated with IPTp3

Variable	Did not receive IPTp3 (*N =* 707)	Received IPTp3 (*N =* 229)	Unadjusted Odds Ratio (OR)
	N (%)	N (%)	OR (95% CI)
**Woman was referred by CHW for at least one ANC visit**			
No	290 (80.1%)	72 (19.9%)	REF
Yes	417 (72.6%)	157 (27.4%)	1.52 (1.10–2.08)**
**Woman’s age (years)**			
14 to 19 years	50 (78.1%)	14 (21.9%)	REF
20 to 24 years	196 (77.8%)	56 (22.2%)	1.02 (0.53–1.98)
25 to 29 years	253 (77.4%)	74 (22.6%)	1.04 (0.55–1.99)
30 to 34 years	140 (74.1%)	49 (25.9%)	1.25 (0.64–2.46)
35 years or more	68 (65.4%)	36 (34.6%)	1.89 (0.92–3.87)
**Parity**			
Nulliparous	107 (77.0%)	32 (23.0%)	REF
Primiparous	134 (70.9%)	55 (29.1%)	1.37 (0.83–2.27)
Multiparous	466 (76.6%)	142 (23.4%)	1.01 (0.66–1.58)
**Malaria education in ANC**			
No	79 (84.0%)	15 (16.0%)	REF
Yes	628 (74.6%)	214 (25.4%)	1.79 (1.01–3.18)*
**Trimester at first ANC visit**			
First trimester	201 (77.3%)	59 (22.7%)	REF
Second trimester	405 (74.2%)	141 (25.8%)	1.19 (0.84–1.68)
Third trimester (7th month)	101 (77.7%)	29 (22.3%)	0.98 (0.59–1.62)

*p < 0.05

**p < 0.01

The mean number of ANC visits and CHW referrals among women who received zero doses were significantly lower than those women who received one or more doses of IPTp (Table 3). In fact, more than half (56.8%) of women in the zero dose group were never referred by a CHW, compared to only 34.5% of women in the one or more dose group. A greater proportion of zero dose women never received malaria-related education during ANC (17.6% versus 8.3%). Finally, a greater proportion attended their first ANC visit during the first trimester amongst the zero dose group than in the 1+ dose group.

**Table 3:** Comparison of women who received IPTp doses versus those who never got IPTp

Variable	One or more doses (*N =* 760)	Zero doses (*N =* 176)	*p*-value for comparison of means by group
	Mean (SD)	Mean (SD)	
**Total number of ANC visits**	2.4 (1.6)	1.7 (1.1)	*p <* 0.01
**Total number of CHW referrals**	0.7 (0.7)	0.4 (0.5)	*p <* 0.01
	N (col %)	N (col %)	
**Woman was referred by CHW for at least one ANC visit**			*p <* 0.01
No	262 (34.5%)	100 (56.8%)	
Yes	498 (65.5%)	76 (43.2%)	
**Woman’s age (years)**			
14 to 19 years	51 (6.7%)	13 (7.4%)	0.769
20 to 24 years	200 (26.3%)	52 (29.5%)	
25 to 29 years	264 (34.7%)	63 (35.8%)	
30 to 34 years	158 (20.8%)	31 (17.6%)	
35 years or more	87 (11.4%)	17 (9.7%)	
**Parity**			0.92
Nulliparous	112 (14.7%)	27 (15.3%)	
Primiparous	152 (20.0%)	37 (21.0%)	
Multiparous	496 (65.3%)	112 (63.6%)	
**Malaria education in ANC**			*p <* 0.01
No	63 (8.3%)	31 (17.6%)	
Yes	697 (91.7%)	145 (82.4%)	
**Trimester at first ANC visit**			*p <* 0.01
First trimester	181 (23.8%)	79 (44.9%)	
Second trimester	473 (62.2%)	73 (41.5%)	
Third trimester (7th month)	106 (13.9%)	24 (13.6%)	
**Women attended four or more ANC visits**			*p <* 0.01
No	604 (79.5%)	160 (90.9%)	
Yes	156 (20.5%)	16 (9.1%)	

**Fig. 3:**
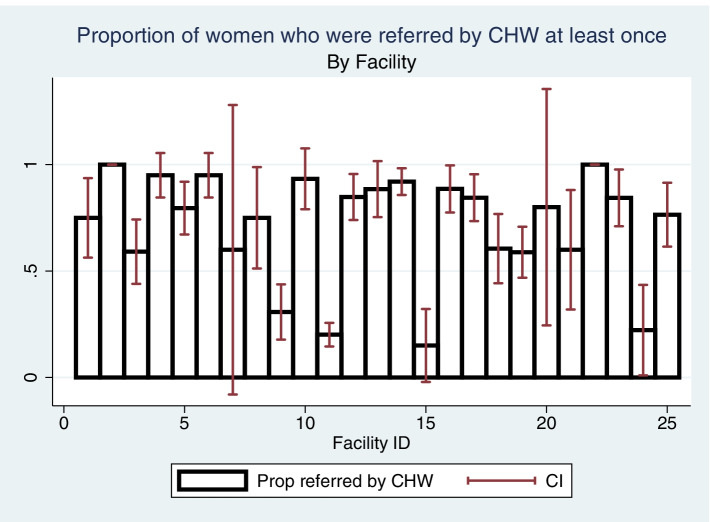
Proportion of women receiving IPTp3, by facility

Figure 3 illustrates the proportion of women who received IPTp3 by facility and corresponding 95% confidence interval. As demonstrated by the blue bars, there is variation across facilities on the proportion of women receiving IPTp3, ranging from 0 to 65%. There is a range of within-facility variation, due in part to the small number of women at certain facilities which results in very wide confidence intervals (for example, facility 20 only has data available for 5 women).

### Fixed effects

Table 4 presents the multilevel logistic regression of characteristics associated with IPTp3. Pregnant women referred for at least one ANC visit by a CHW had 1.60 times greater odds of IPTp3 than women who were never referred. The odds of IPTp3 increased with each age category compared to the reference category (14–19 years), however, only the comparison of women ages 35 and older to those 14–19 years of age (reference category) was statistically significant. After adjusting for covariates and clustering, receipt of malaria education during ANC was associated with a 51% increase in the odds of IPTp3, but this was not statistically significant.

**Table 4:** Multilevel logistic regression of characteristics associated with IPTp3

Variable	Empty model	Full model
**Fixed effects**		**AOR (95% CI)**
**Woman was referred by CHW for at least one ANC visit**		
No		REF
Yes		1.60 (1.08–2.38)**
**Woman’s age (years)**		
14 to 19 years		REF
20 to 24 years		1.03 (0.50–2.11)
25 to 29 years		1.27 (0.61–2.67)
30 to 34 years		1.69 (0.76–3.72)
35 years or more		2.35 (1.01–5.44)*
**Parity**		
Nulliparous		REF
Primiparous		1.26 (0.73–2.18)
Multiparous		0.75 (0.44–1.28)
**Malaria education in ANC**		
No		REF
Yes		1.51 (0.80–2.82)
**Trimester at first ANC visit**		
First trimester		REF
Second trimester		1.24 (0.86–1.80)
Third trimester (7th month)		1.10 (0.65–1.88)
**Random Effects**		
Variance (constant)	0.554 (0.21–1.45)	0.519 (0.20–1.38)
ICC	0.144 (0.061–0.305)	0.136 (0.06–0.30)
AIC	1020.19	1018.53
MOR	2.03	1.99

*p < 0.05

**p < 0.01

### Random effects

The null model showed that 14.4% of the variation in IPTp3 in the study population was attributed to unobserved facility characteristics. The variance of the constant also shows a significant amount of variation between the overall intercept and facility-specific intercepts (variance = 0.554, 95% CI: 0.21–1.45). The median odds ratio (MOR) indicates that if a woman moved to a facility with higher odds of IPTp3, the odds of IPTp3 receipt increased by two-fold.

## Discussion

This study used a unique data source, the maternity record book, to assess whether a referral to antenatal care by a community health worker was associated with IPTp3 uptake. This is of high importance in sub-Saharan Africa, where health systems face both human resource and financial constraints. However, the low uptake of IPTp3 reported in this study, which is similar to that reported in some sub-Saharan African subregions, [11–13] indicates that more improvements in coverage must be made in order to reach the Sustainable Development Goal Target 3.3, to end the malaria epidemic (in addition to the AIDS and tuberculosis epidemics). We found that pregnant women who were referred to ANC by a CHW had 1.6 times greater odds of completing IPTp3. Furthermore, there were no differences in referrals by maternal characteristics, indicating that the CHWs applied the referral procedures equally, rather than to selective groups of women. Our findings also suggest that receiving IPTp from a CHW did not disrupt ANC attendance or result in lower ANC attendance. This further strengthens the evidence base for the continued scale-up of community-directed interventions to close the gap between ANC attendance and IPTp3 uptake.

Although they have no medical training, CHWs have potential to improve access to maternal and child health interventions, including IPTp [14]. This study demonstrated that women referred by CHWs were more likely to receive IPTp3 than those who were not referred. Previous studies in Nigeria, Burkina Faso, and Malawi have shown that the implementation of community-based IPTp increased coverage of two or three doses (dependent on study) of IPTp [8, 9, 15, 16]. Similar to the findings in Nigeria and Burkina Faso, C-IPTp did not distract from ANC attendance in this setting; there were no differences in the number of ANC visits between women who received community distributed IPTp and those who only received IPTp at a facility. This is different from the findings in Malawi, where the attendance of at least two ANC visits dropped significantly in the intervention villages (87.3 to 51.5% of women). The authors hypothesized that this could be due to health systems issues (efficiency and professionalism of care), which resulted in women preferentially accessing care through the CHWs rather than at the facilities. It is possible we did not see the same impact on ANC attendance due to the different setting and health systems, which did not present the same deterrents to care. Likewise, a critical component of the approach in TIPTOP was a focus on ANC, including refresher trainings for facility-based providers, engagement of facilities in supporting and mentoring CHWs, and strengthening linkages between communities and facilities. These parts of our strategy likely contributed to C-IPTp not having a negative impact on ANC attendance. A qualitative study in Burkina Faso found that with proper training and supervision, the delivery of IPTp by CHWs was both feasible and acceptable as reported by facility-based health workers and CHWs [17]. Taken together, the quantitative and qualitative evidence supports continued scaling up of the C-IPTp model.

There were significant differences in IPTp3 coverage by health facility, where the proportion of women receiving IPTp3 ranged from 0 to 65%. Nearly 15% of the variation in the IPTp3 outcome was attributed to unobserved facility characteristics. Other studies have outlined numerous health-system level barriers to IPTp uptake, including stock outs, increased fees for SP doses, distance to the facility, long waiting times at the facility, unclear IPTp protocols and insufficient provider training [18–21]. It’s likely that these barriers affected the 25 facilities in our sample to varying degrees, though unfortunately we were unable to assess their impact in our study. Further research that elucidates whether and how the community distribution of IPTp model mitigates these barriers would be beneficial for future programming and policy efforts across sub-Saharan Africa.

In our study, timing of the first ANC visit was not significantly associated with IPTp3 receipt. Unlike other studies that have shown that earlier attendance results in a higher likelihood of IPTp receipt [18, 22, 23], the trend in our population showed slightly increased odds of IPTp3 for women who presented in the second trimester compared to the first, though these were not statistically significant. A possible explanation is that because IPTp is initiated as early as possible in the second trimester, if a woman that initiates ANC in the first trimester but does not return for the appropriate number of visits, she may in fact have fewer opportunities to receive SP than a woman who presented in the second trimester and attended the same number of visits. In our study, only 18.38% of women attended four or more antenatal care visits, which may have played a role in the trend described above. This proportion is far lower than the most recent Demographic and Health Survey estimate of 57% of women attending four or more ANC visits [4]. A possible explanation for the discrepancy is the DHS collects antenatal care data by maternal report of her most recent live birth in the last 5 years, which introduces the possibility of recall and social desirability bias. This may result in an overestimation of the number of visits, if the woman believes that reported a greater number of visits would be viewed as a favorable response by the interviewer [24]. This potential bias would not apply to our data source, the MRB, as it is completed by health care workers in real time. An alternate explanation, given the missing data for other variables in the analysis, is that the MRB data is incomplete, resulting in an underestimation of the number of ANC visits a woman attended. We also found that women who received malaria-related education during ANC were more likely to receive IPTp3, but this was only significant in the bivariate model. This aligns well with other studies that have demonstrated that knowledge about IPTp and the risk of malaria during pregnancy is associated with increased uptake of IPTp [18, 25]. Unlike other studies the present study was unable to explore the effect of socioeconomic status, education level and marital status on uptake of IPTp due to missing data.

An aspect of the intervention that was not captured by the MRB was the counseling on the benefits of ANC by the CHW to the pregnant woman prior to administering the referral, which likely was a key motivator for ANC attendance. A recent meta-analysis reported that CHW interventions, including counseling, increased knowledge and ANC utilization amongst pregnant women [26]. For future research, it would be beneficial to capture the counseling component in addition to the referral, to examine the role of counseling by CHWs and whether there it results in a difference in ANC attendance and IPTp uptake.

A strength of this study is the use of the data source, the maternal record booklet, which allowed for individual-level data that covered the course of the woman’s pregnancy. To our knowledge, this is the first time MRB data were used outside of clinical care. This allowed for the analysis of individual-level, regularly collected data, which is not available in more standard HMIS data sources where the data are only in aggregate and that avoids the potential limitations and biases from household surveys, as referenced earlier. In line with the global recognition of the importance of strengthening routine health information systems, the MRB source must be strengthened as well for future research and programmatic efforts, as the high missingness of maternal characteristic data indicates there are some quality issues. A second strength of this study is that we used a multi-level model to account for facility-level differences, which produces more robust effect estimates. A limitation of the analysis is that although the outcome and primary covariate data recorded in the MRB were complete, as mentioned earlier, a substantial number of observations had missing information for maternal characteristics (e.g., education, occupation) that did not allow for their inclusion in the analysis. A second limitation is that the MRB data source did not allow for the inclusion of facility characteristics in the model, such as availability of trained providers or facility readiness, which could have an impact on IPTp3. A final limitation is that there may be some selection bias, as we could not visit all sites initially considered due to instability issues, which could mean that those women and facilities included in the analysis are systematically different from those excluded, limiting the generalizability of the results.

## Conclusion and recommendations

This study found that CHW referral was associated with higher odds of IPTp3, that CHWs were able to apply the referral procedures equally and that the C-IPTp model did not reduce regular ANC attendance, further strengthening the evidence based for CHW delivered interventions. Future research should explore barriers and facilitators of CHWs interventions to best inform scale-up of these programs. CHWs should be recognized, trained and incentivized as important cadres in the health systems in these regions in order to build their competence and improve health outcomes for mothers and their newborn.

## Supplementary Information

**Additional file 1.**

## Data Availability

The datasets used and/or analyzed during the current study are included in this published article as an Excel spread sheet/Stata (and its supplementary information files). In addition, it is available from the corresponding author on reasonable request.

## Abbreviations

ANC: Antenatal care
aOR: Adjusted Odds Ratio
aPR: Adjusted Prevalence Ratio
CHW: Community health worker
C-IPTp: Community distribution of IPTp
IPTp: Intermittent preventive treatment for malaria during pregnancy
IPTp3: Three or more doses of IPTp
ITN: Insecticide treated bed nets
LGA: Local government area
MRB: Maternity Record Booklet
SD: Standard Deviation
TIPTOP: Transforming Intermittent Preventive Treatment for Optimal Pregnancy
VIF: Variance Inflation Factor
WHO: World Health Organization
