# Supplementary material for: Retrospective evaluation of referral by community health workers on the uptake of intermittent preventive treatment of Malaria in pregnancy in Ohaukwu, Ebonyi State, Nigeria
Source: BMC Pregnancy Childbirth. 2022 Jul 27;22:599. doi: 10.1186/s12884-022-04921-7 (PMC9327266; doi:10.1186/s12884-022-04921-7)
Supplement: Supplementary file 1 — Additional file 1. [file 12884_2022_4921_MOESM1_ESM.docx]

**Supplementary materials**

**Supplementary Figure 1. Age distribution**

**
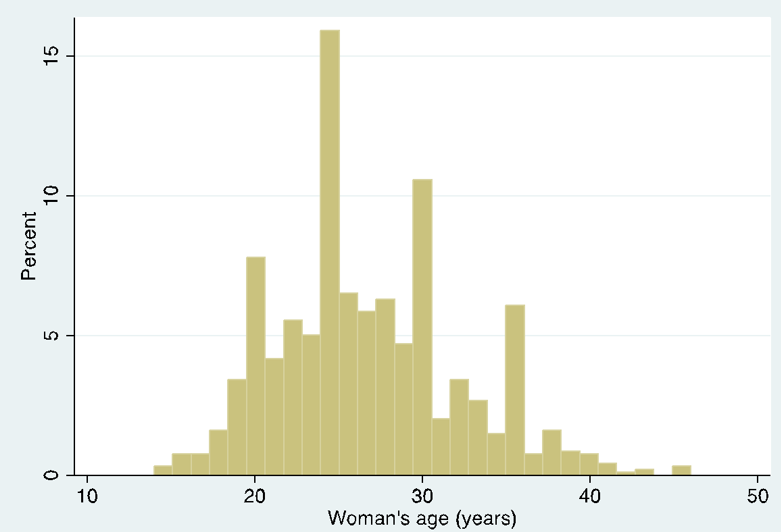
**

Note: Mean age= 26.79 (SD=5.49)

**Supplementary Figure 2. Distribution of the # of ANC visits**

**
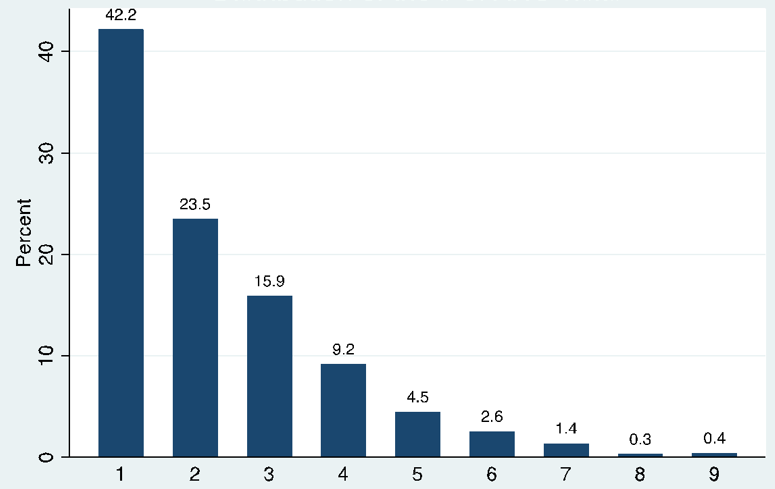
**

Note: Mean # visits = 2.28 (SD=1.54)

**Supplementary Table 1. Multilevel Poisson regression of characteristics associated with CHW referral**

| **Variable** | **Empty model** | **Full model** |
| --- | --- | --- |
| **Fixed effects** |  | **aPR (95% CI)** |
| **Woman's age (years)** |  |  |
| 14 to 19 years |  | REF |
| 20 to 24 years |  | 1.03 (0.83-1.30) |
| 25 to 29 years |  | 1.10 (0.87-1.38) |
| 30 to 34 years |  | 1.08 (0.84-1.38) |
| 35 years or more |  | 1.03 (0.76-1.39) |
| **Parity** |  |  |
| Nulliparous |  | REF |
| Primiparous |  | 1.00 (0.85-1.18) |
| Multiparous |  | 1.02 (0.84-1.25) |
| **Trimester at first ANC visit** |  |  |
| First trimester |  | REF |
| Second trimester |  | 1.09 (0.92-1.29) |
| Third trimester (7th month) |  | 0.97 (0.87-1.08) |
| **Random Effects** |  |  |
| Variance (constant) | 0.149 (0.05-0.47) | 0.160 (0.05-0.48) |

*p<0.05, **p<0.01
